# Pre-therapy serum albumin-to-globulin ratio in patients treated with neoadjuvant chemotherapy and radical nephroureterectomy for upper tract urothelial carcinoma

**DOI:** 10.1007/s00345-020-03479-3

**Published:** 2020-10-16

**Authors:** Benjamin Pradere, David D’Andrea, Victor M. Schuettfort, Beat Foerster, Fahad Quhal, Keiichiro Mori, Mohammad Abufaraj, Vitaly Margulis, Marine Deuker, Alberto Briganti, Tim Muilwijk, Kees Hendricksen, Yair Lotan, Pierre Karakiewic, Shahrokh F.Shariat, For the UTUC collaboration

**Affiliations:** 1 Department of Urology, Comprehensive Cancer Center, Vienna General Hospital, Medical University of Vienna, Währinger Gürtel 18-20, 1090 Vienna, Austria; 2 Department of Urology, University Hospital of Tours, Tours, France; 3 Department of Urology, Kantonsspital Winterthur, Winterthur, Switzerland; 4 Department of Urology, King Fahad Specialist Hospital, Ad Dammām, Saudi Arabia; 5 Department of Urology, The Jikei University School of Medicine, Tokyo, Japan; 6 Department of Special Surgery, Jordan University Hospital, The University of Jordan, Amman, Jordan; 7 Department of Urology, University of Texas Southwestern Medical Center, Dallas, TX USA; 8 Cancer Prognostics and Health Outcomes Unit, University of Montreal Health Center, Montreal, Canada; 9 Department of Urology, University Hospital Frankfurt, Frankfurt, Germany; 10 Department of Urology, Urological Research Institute, Vita-Salute University, San Raffaele Scientific Institute, Milan, Italy; 11 Department of Urology, University Hospitals Leuven, Leuven, Belgium; 12 Department of Urology, The Netherlands Cancer Institute-Antoni Van Leeuwenhoek Hospital, Amsterdam, The Netherlands; 13 Department of Urology, University of Texas Southwestern Medical Center, Dallas, TX USA; 14 Department of Urology, Weill Cornell Medical College, New York, NY USA; 15 Department of Urology, Second Faculty of Medicine, Charles University, Prague, Czech Republic; 16 Karl Landsteiner Institute of Urology and Andrology, Vienna, Austria; 17 Division of Urology, Department of Special Surgery, Jordan University Hospital, The University of Jordan, Amman, Jordan; 18 European Association of Urology Research Foundation, Arnhem, The Netherlands

**Keywords:** AGR, UTUC, Neoadjuvant, Urothelial, Biomarker

## Abstract

**Purpose:**

The accurate selection of patients who are most likely to benefit from neoadjuvant chemotherapy is an important challenge in oncology. Serum AGR has been found to be associated with oncological outcomes in various malignancies. We assessed the association of pre-therapy serum albumin-to-globulin ratio (AGR) with pathologic response and oncological outcomes in patients treated with neoadjuvant platin-based chemotherapy followed by radical nephroureterectomy (RNU) for clinically non-metastatic UTUC.

**Methods:**

We retrospectively included all clinically non-metastatic patients from a multicentric database who had neoadjuvant platin-based chemotherapy and RNU for UTUC. After assessing the pretreatment AGR cut‐off value, we found 1.42 to have the maximum Youden index value. The overall population was therefore divided into two AGR groups using this cut‐off (low, < 1.42 vs high, ≥ 1.42)*.* A logistic regression was performed to measure the association with pathologic response after NAC. Univariable and multivariable Cox regression analyses tested the association of AGR with OS and RFS.

**Results:**

Of 172 patients, 58 (34%) patients had an AGR < 1.42. Median follow-up was 26 (IQR 11–56) months. In logistic regression, low AGR was not associated with pathologic response. On univariable analyses, pre-therapy serum AGR was neither associated with OS HR 1.15 (95% CI 0.77–1.74; *p* = 0.47) nor RFS HR 1.48 (95% CI 0.98–1.22; *p* = 0.06). These results remained true regardless of the response to NAC.

**Conclusion:**

Pre-therapy low serum AGR before NAC followed by RNU for clinically high-risk UTUC was not associated with pathological response or long-term oncological outcomes. Biomarkers that can complement clinical factors in UTUC are needed as clinical staging and risk stratification are still suboptimal leading to both over and under treatment despite the availability of effective therapies.

## Introduction

Upper Tract Urothelial Carcinoma (UTUC) is a rare disease which represents only 5% of urothelial carcinoma, and has generally a worse prognosis compared to bladder cancer [1]. Surgery is the cornerstone of its management and its indication is based on a risk stratification model to choose between radical nephroureterectomy (RNU) and kidney sparing surgery (KSS) [2–5]. Based on the management of bladder cancer, it has been suggested to offer perioperative chemotherapy to patients at high risk [6–8]. Recently, a phase 3 prospective trial reported a benefit in terms of disease-free survival and metastasis-free survival for adjuvant platinum-based chemotherapy for UTUC patients with pT2 and higher stage after RNU [9]. Optimal adjuvant systemic treatment after RNU is, however, not deliverable to many patients due to their loss of renal function with RNU in this elderly population. On the other hand, there is no solid evidence for neoadjuvant systemic treatment in UTUC with only small retrospective studies [10]. With the reliability and reproducibility of the current preoperative risk stratification being poor, there is a high risk of both overtreatment and undertreatment [2, 11]. Second, giving NAC to patients with UTUC who are generally older with comorbidities may lead to significant adverse events.

Nevertheless, despite the weakness of available studies, a recent multicenter study including 267 patients reported that pathologic complete response was achieved in 10% of patients and downstaging in 45%, resulting in improved survival [12]. In addition, a metanalysis still found a benefit for NAC in UTUC with an improvement in pathologic downstaging (pDS) and, pathological complete response (pCR) as well as on overall (OS) and cancer-specific (CSS) survival [6]. While we are still waiting for the results of the first randomized trial assessing the benefit of NAC (URANUS NCT02969083) in UTUC, the biggest challenge is to identify which patients are most likely to benefit from preoperative systemic therapy and which patient can be spared an inefficient NAC and, therefore, be offered a different systemic therapy or RNU alone with or without adjuvant chemotherapy.

Nowadays, molecular signatures arising from genetic sequencing are actually under investigation [13–15], but simple and cost-effective biomarkers such as serum protein-based biomarkers could still be useful as long as they add value beyond that obtained by standard features [16, 17].

Serum albumin-to-globulin ratio (AGR) has been shown to prognosticate oncological outcomes for many malignancies such as bladder cancer and UTUC [18–23]. Albumin and globulins are the two major serum proteins and have been proven to reflect the inflammatory process [24]. Albumin has an antioxidative role in plasma and in the interstitial space allowing the matrix deposition and cell proliferation. During inflammation, an hypoalbuminemia is induced due to the increased capillary escape of serum albumin into the interstitium. Therefore, serum albumin can be classified as a negative acute-phase protein. Globulin contains inflammatory mediators such as chemokines, cytokines, and other small inflammatory proteins. The local or systemic immune response in cancer-related inflammation is associated with an increased production of these inflammatory mediators. The combination of these proteins as a ratio can help to assess the systemic inflammatory response and the patient nutritional status [25]. In UTUC, abnormal serum preoperative AGR has been found to be associated with adverse pathologic features, survival, and poorer outcomes in patients treated with RNU [21, 22]. To date, the assessment of serum AGR in UTUC has been only performed for patients without NAC, and only one study in rectal cancer tried to evaluate its predictive value before an NAC with capecitabine or 5-FU based chemotherapy, but did not found a predictive value of AGR in this indication [26].

We hypothesized that pre-therapy serum AGR could be a predictor in this setting. To test this, we assessed the value of pre-therapy serum AGR for predicting pathological response and its prognostic value for survival outcomes in a large multicentric international cohort of contemporary patients who received platinum-based NAC before RNU for UTUC.

## Materials and methods

### Study population

We performed a retrospective analysis of patient treated with NAC followed by radical nephroureterectomy (RNU) for UTUC from an multicenter database arising from international cooperation. Patients with clinically distant metastatic disease (M status), positive clinical lymph nodes (cN+), and those lost to follow-up were not included in the analysis. The study was approved by the local committees of ethics in all institutions and informed consent was obtained from eligible patients. Patient information was anonymized prior to data sharing.

### Management and follow-up

NAC regimens consisted of platin-based combination chemotherapy if the renal function allowed it [7]. Chemotherapy regimen (cisplatin or carboplatin) and number of cycles were administered at clinician discretion in accordance with institutional standards and based on individual shared decision-making with the patient.

All RNU procedures were performed using standard techniques [5, 27, 28]. The decision to perform lymphadenectomy and its extent were at the surgeon discretion based on patient and preoperative disease characteristics following standard templates previously described [29]. All surgical specimens were exanimated by a local dedicated uro-pathologists. Tumor grade was determined on the basis of the World Health Organization/International Society of Urologic Pathology classification of 2004 [30]. Tumor stage was evaluated using the 2002 Union for International Cancer Control tumor, node, metastasis classification system.

Preoperative baseline blood tests were performed within the month prior to RNU. Serum AGR levels were calculated as the ratio of baseline serum albumin to the total protein-baseline serum albumin. To define the optimal pretreatment AGR cut‐off value, we carried out a time‐dependent receiver-operating characteristic curve analysis for 3-year OS as the end‐point, considering the median OS time (12 months). The median value of AGR was calculated as 1.57 (IQR 1.37–1.83). A cut-off value 1.42 was determined as the highest Youden index value [21, 22]. Therefore, we divided the population into two groups according to this AGR cut-off (lower < 1.42 vs higher ≥ 1.42). OS time was calculated from the date of NAC to death or last follow-up. CSS time was calculated from the date of the NAC to death from disease or last follow-up.

The patient follow-up was performed according to guidelines, at the time, with a consultation generally every 3 months during the first 2 years after RNU, every 6 months the third to the fifth year, and then annually.

### Outcome measurement

Our primary objective was to evaluate the association of pre-therapy serum AGR with pathologic response after NAC assessed based on the RNU specimen. Pathologic responses were defined as pathologic complete response (ypT0) and pathologic downstaging (≤ ypT1).

Our secondary objective was to evaluate the association of serum AGR with oncologic survival outcomes including recurrence-free (RFS) and overall (OS) survival.

### Statistical analysis

The differences between continuous and categorical variables across AGR groups were assessed using Mann–Whitney *U* test and Chi-square tests, respectively. To assess the relation between groups and pathologic outcomes, we used binary univariable and multivariable logistic regression analyses. A log-rank test was performed to compare differences in survival between AGR groups, Kaplan–Meier curves were used to estimate RFS and OS. Univariable and multivariable Cox regression analyses were performed to determine the factors associated with RFS, CSS, and OS. We included in the model the multivariable analysis of all the variables with *p* value < 0.2 in the univariable analysis as well as the most clinically relevant variables according to the primary endpoint. Statistical significance was set at *p* < 0.05. All tests were two-sided. Analyses were performed using R version 3.6.2. (2009–2019 RStudio, Inc.).

## Results

Overall, 172 patients were included in the analyses. Among them, 58 (34%) patients had an AGR < 1.42 (low AGR) and 114 (66%) had an AGR ≥ 1.42 (high AGR). Patient’s characteristics according to their serum AGR category are shown in Table 1. There was no significant difference between groups. Pathologic characteristics after NAC and RNU were also similar between groups (Table 2). There were 20 patients with a pT0 on the final specimen, 10 (16.4) in the high AGR and 10 (33.3) in the low AGR group.

**Table 1:** Patients’ characteristics according to the pretherapy serum AGR level status (high vs. low) in patients treated with neoadjuvant chemotherapy before radical nephroureterectomy for upper tract urothelial carcinoma

	All	High (≥ 1.42)	Low (< 1.42)	*p*
*n*	172	114	58	
Age (median [IQR])	68.00 [63.00, 73.00]	68.00 [63.00, 73.00]	67.50 [63.50, 73.75]	0.95
Gender male (%)	122 (70.9)	86 (75.4)	36 (62.1)	0.10
BMI (mean (SD))	27.4 (5.6)	27.6 (5.8)	26.9 (5.0)	0.47
Previous history of bladder cancer (%)	60 (34.9)	38 (33.6)	22 (38.6)	0.64
High grade on cytology (%)	97 (56.4)	60 (77.9)	37 (88.1)	0.26
Multifocality (%)	33 (19.2)	21 (20.0)	12 (21.8)	0.95
Hydronephrosis (%)	67 (39.0)	40 (35.4)	27 (47.4)	0.18
Neoadjuvant treatment regimen (%)				1.00
Cisplatin-based	162 (94.2)	107 (93.9)	55 (94.8)	
Carboplatin-based	10 (5.8)	7 (6.1)	3 (5.2)

**Table 2:** Pathologic characteristics after RNU according to the pretherapy serum AGR level status (high vs. low) in patients treated with neaoadjuvant chemotherapy before radical nephroureterectomy for upper tract urothelial carcinoma

	All	High (≥ 1.42)	Low (< 1.42)	*p*
Pathologic stage (%)				0.13
pT0	20 (11.6)	10 (16.4)	10 (33.3)	
pTa	28 (16.3)	23 (37.7)	5 (16.7)	
pTis	11 (6.4)	7 (11.5)	4 (13.3)	
pT1	32 (18.6)	21 (34.4)	11 (36.7)	
Specimen ≥ pT2 (%)	80 (46.5)	52 (46.0)	28 (48.3)	0.91
Pathologic high grade (%)	140 (81.4)	93 (92.1)	47 (97.9)	0.30
Pathologic positive lymph nodes (%)	33 (19.2)	22 (19.3)	11 (19.0)	1.00
Positive surgical margin (%)	10 (5.8)	6 (5.4)	4 (7.3)	0.89
Overall pathologic response rate (%)	87 (50.6)	59 (51.8)	28 (48.3)	0.79
Pathologic complete response rate (%)	18 (10.5)	10 (8.8)	8 (13.8)	0.45

A logistic regression analysis was performed to assess if AGR was predictor of overall or complete pathologic response. In univariable analysis, a low serum AGR was not associated with pathologic downstaging (≤ ypT1N0) [OR 0.87, (95% CI 0.46–1.63); *p* = 0.66]. On multivariable logistic regression analysis that adjusted for the effect age, gender, and tumor location, pre-therapy AGR was still not correlated with partial or complete pathologic response (all *p* value > 0.05).

**Fig. 1:**
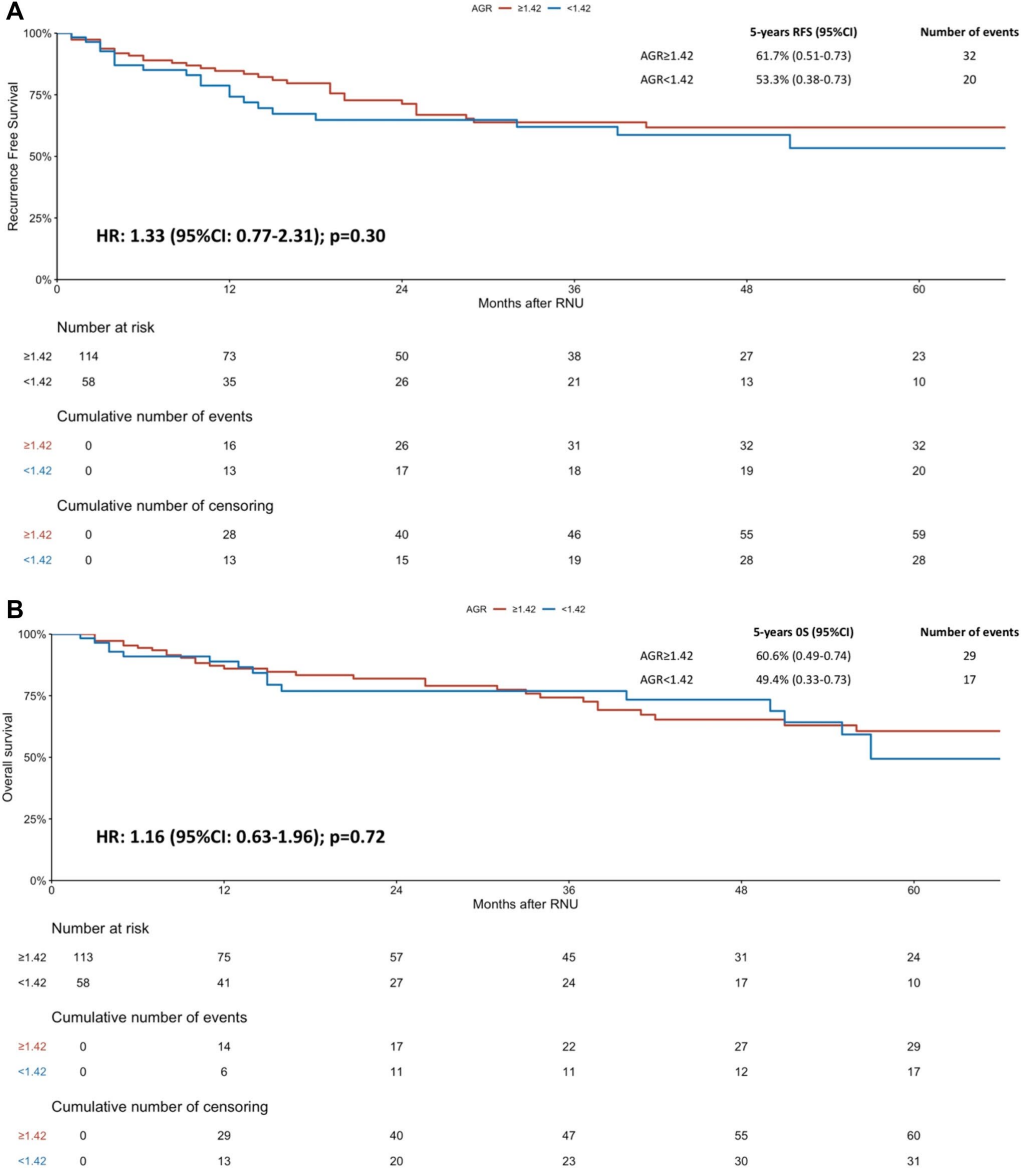
Kaplan–Meier analysis for recurrence-free survival (RFS) **a** and overall survival (OS), **b** stratified by albumin globulin ratio (AGR) at a cut-off of 1.42 in 172 patients treated with neoadjuvant chemotherapy followed by radical nephroureterectomy for upper tract urothelial cancer (UTUC)

The median follow-up for the whole cohort was 26 (IQR 11–56) months. The 5-year RFS estimates were 61.7% for high AGR and 53.3% for low AGR. There was no difference in the Kaplan–Meier analysis according to the serum AGR for RFS [HR 1.33, 95% CI 0.77–2.31; *p* = 0.30] (Fig. 1a). The 5-year OS estimates were 60.6% for high AGR and 49.4% for low AGR. There was no difference in OS on the Kaplan–Meier analysis according to AGR category [HR 1.16, 95% CI 0.63–1.96; *p* = 0.72] (Fig. 1b).

On univariable analyses, the only variables associated with RFS and OS were advanced tumor stage (≥ pT2) on pathological examination (*p* < 0.001), positive lymph nodes (*p* < 0.001), response to NAC on final pathologic examination (*p* < 0.001), and cisplatin-based regimen (*p* = 0.01) (Table 3).

**Table 3:** Univariable Cox regression analyses on recurrence-free survival and overall survival in patients treated with neaoadjuvant chemotherapy before radical nephroureterectomy for upper tract urothelial carcinoma

Variable	Recurrence-free survival (RFS)			Overall survival (OS)		
	HR	95% CI	*p* value	HR	95% CI	*p* value
Age	1.02	0.99–1.05	0.18	1.06	1.02–1.09	**0.016**
Sex: female Male	Ref	Ref	–	Ref	Ref	–
	0.72	0.40–1.25	0.24	0.65	0.37–1.14	0.13
Previous BCa	0.84	0.46–1.53	0.57	1.072	0.60–1.90	0.81
Hydronephrosis	1.36	0.89–2.06	0.15	1.14	0.62–2.08	0.66
Multifocality	1.09	0.43–1.86	0.89	0.83	0.40–1.71	0.62
AGR low	1.34	0.77–2.31	0.3	1.16	0.63–1.95	0.70
NAC regimen cisplatine-based	0.32	0.13–0.82	**0.01**	0.41	0.15–1.17	0.09
pTNM pT ≥ 2	5.94	3.11–11.3	**< 0.001**	2.34	1.32–4.14	**< 0.001**
pTNM pN +	4.03	2.33–6.97	**< 0.001**	2.65	1.77–3.97	**< 0.001**
Overall pathological response	0.17	0.09–0.34	**< 0.001**	0.42	0.24–0.74	**0.002**
Pathological complete response	0.29	0.07–1.20	0.08	0.74	0.29–1.87	0.53

Bold indicates statistically significant

**Fig. 2:**
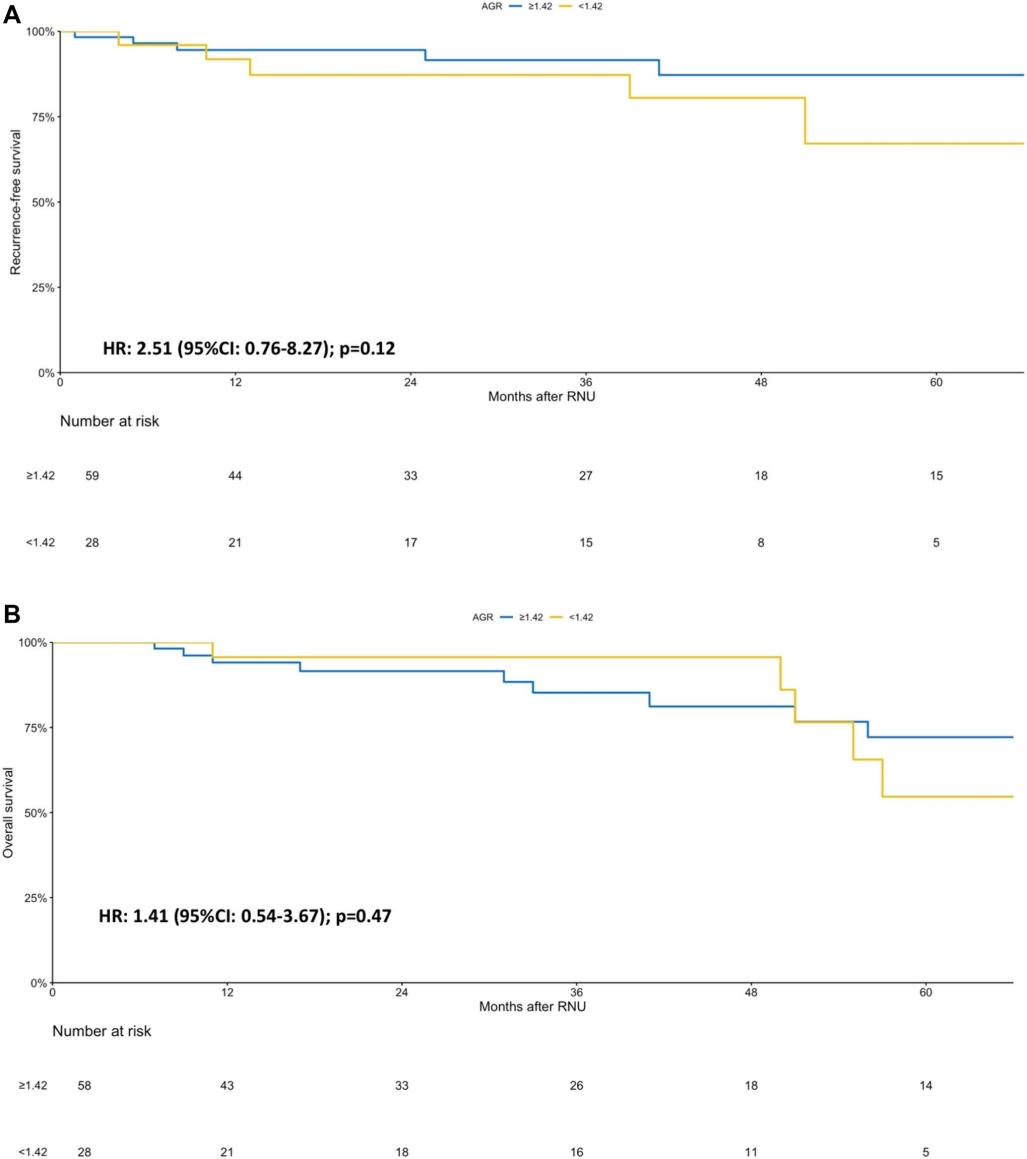
Sub-group Kaplan–Meier analysis for recurrence-free survival (RFS) (**a**) and overall survival (OS) (**b**) stratified by albumin globulin ratio (AGR) at a cut-off of 1.42 in patients who responded to neoadjuvant chemotherapy

**Fig. 3:**
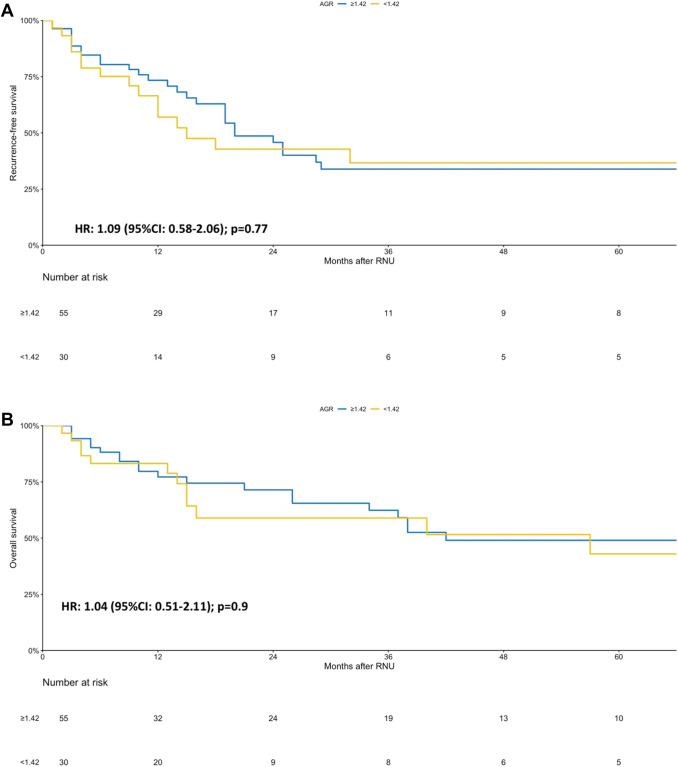
Sub-group Kaplan–Meier analysis for recurrence-free survival (RFS) (**a**) and overall survival (OS) (**b**) stratified by albumin globulin ratio (AGR) at a cut-off of 1.42 in patients who did not respond to neoadjuvant chemotherapy

A predefined subgroup analysis was performed into responders and nonresponders of NAC. The AGR level before NAC was not correlated with OS or RFS (all *p* value > 0.05) in both of these subgroups (Figs. 2, 3). Similarly, in the subgroup analysis of patient with complete response only, AGR was still not associated with RFS or OS (all *p* value > 0.05).

We also performed a subgroup analysis according to the regimen of NAC. Neither in the group of patients who received cisplatin-based chemotherapy nor in the group of patients who received carboplatin-based chemotherapy was AGR associated with OS, RFS, or CSS (all *p* value > 0.05).

## Discussion

This study intended to assess the potential role of serum AGR as a biomarker to predict pathological response and oncological outcomes in patients treated by NAC followed by RNU. The Cox regression analysis did not identify any significant relationship between pre-therapy serum AGR and oncological outcomes. Moreover, we assessed if the AGR status could predict response to NAC using standard logistic regression analyses. Again, pre-therapy serum AGR was not correlated to NAC. We confirmed the association of established clinicopathological predictors of oncologic and pathologic outcomes.

These findings are not in contrast with the current literature. In fact, two studies already assessed the potential of the serum AGR as a biomarker in patients with UTUC treated by RNU. In 2015, Zang et al. found that an AGR level below 1.45 was an independent predictor of OS and CSS in a retrospective cohort of 187 patients treated by RNU [22]. Similarly, Xu et al. found in 620 patients that a low AGR level was associated with OS CSS and also with RFS using the same cut-off [21]. Despite these positives and promising results, none of these studies assessed the serum AGR status using Harrel’s concordance index (C-index) or decision curve analysis (DCA) to confirm the clinical relevance of the biomarker [16]. Therefore, serum AGR still needs further evaluation and validation in larger prospective cohorts. Moreover, in these studies, all the patients who had an NAC were excluded from the analysis. Thus, our study is the first to explore the potential value of pretherapy serum AGR in this specific population of patients who received NAC.

Despite a larger number of studies exploring the role of AGR as an oncological biomarker in different cancers, few studies assessed its value for predicting response to NAC before surgery. Indeed, only one study, in rectal cancer, assessed specifically AGR in patients receiving NAC before surgery and found a weak but statistically significant association with pathological and oncological outcomes [31]. Nevertheless, many other prognostic serum-based biomarkers have been assessed before NAC, but their prognostic value in this specific setting remains unclear. For example, the neutrophil-to-lymphocyte ratio (NLR) which has been largely assessed, did not predict complete response to NAC despite its good predictive value on oncological outcome in patients without NAC in other cancers such as breast, esophageal [32], or cervical cancer [33] [34, 35]. It was only a potential predictor in association with other markers such as the platelet-to-lymphocyte ratio [35]. Similarly in urothelial carcinoma, the benefit of NLR in NAC setting is controversial and Seah et al. did not find association with pathologic response in MIBC, but Brisan et al. found a slight association between NLR and response to NAC in patients with MIBC only when there were squamous cell features [36].

The hypothesis for these controversial results is mostly based on the inflammatory and nutritional status alteration during NAC. Many studies found a high heterogeneity and modifications in inflammatory biomarkers during and after NAC [19, 37]. The modification between pre- and post-NAC of these biomarkers implied that their clinical value might be complicated to interpret. Interestingly, some studies found that post-NAC inflammatory biomarkers could not predict survival [38]. Moreover, very few studies tried to assess the inflammatory markers changes during NAC and their impact on the prognosis, and were not able to give a clear conclusion [25, 39]. Chemotherapy and surgery impact inflammation and immunologic status, and their combination might also modify largely the serum biomarkers, leading to these controversial results.

Several limitations of our study should be acknowledged. The main limitation of the study was its retrospective and multicenter design which led to not standardized laboratory or pathologic evaluation that could confound the results. AGR was assessed only before NAC, but it should have been interesting to evaluate it also after chemotherapy. Its combination with other serum biomarkers might have been interesting to test, but due to the retrospective and multicentric design, we were not able to assess other serum variables. There is a potential intervention bias in our observational cohort study from the inequality in nutritional support. The malnutrition might have been corrected temporarily to meet requirements for NAC and surgery, and the markers might not reflect the status of the autonomic nutrition level.

## Conclusion

In our study, pretherapy serum AGR status before NAC followed by RNU was neither associated with pathologic response nor oncological outcomes on RFS and OS. Advanced stage, positive lymph nodes, and response to NAC were associated with RFS and OS.

The potential effect of NAC on systemic inflammation in UTUC patients, the tumor microenvironment, and the clinical natural history of the cancer may explain the lack of predictive accuracy for serum AGR biomarker in this specific setting.

## Data Availability

Yes.

## Code availability

Yes.
